# Clinical and Laboratory Findings of 12 Children with Invasive Meningococcal Disease in Pediatric Intensive Care Unit

**DOI:** 10.1155/2021/9713918

**Published:** 2021-09-04

**Authors:** Eylem Kiral, Ayse Filiz Yetimakman

**Affiliations:** ^1^Department of Pediatric Intensive Care, Eskisehir Osmangazi University Faculty of Medicine, Eskisehir 26040, Turkey; ^2^Department of Pediatric Intensive Care, Kocaeli University Faculty of Medicine, Umuttepe, Izmit 41001, Turkey

## Abstract

**Introduction:**

Invasive meningococcal disease (IMD) is a serious infectious disease requiring stay in a pediatric intensive care unit (PICU) that continues to be associated with high morbidity and mortality rates. Prompt recognition, early antibiotic therapy, and aggressive supportive therapies can reduce mortality. We aimed to assess the clinical and laboratory characteristics of children with IMD. *Patients and Methods*. We retrospectively evaluated the medical records of 12 children with IMD requiring PICU stay between January 2018 and July 2019.

**Results:**

We followed up 12 patients (five girls and seven boys, 5–168 months of age, and four below one year of age) with IMD (nine patients have meningococcemia with meningitis, and three patients have meningococcemia) in PICU. All children were previously healthy and have not received meningococcal vaccines. Their pediatric risk of mortality (PRISM) scores varies between 5 and 37, four of the patients required mechanical ventilation, and the predicted mortality was 39% at admission. Seven patients had catecholamine refractory septic shock and disseminated intravascular coagulation (DIC). Three of the patients required extracorporeal treatment. The predominant serogroup is Men B (5/12). The mortality rate was 16.6% with early use of antibiotics, fluids, and other interventions.

**Conclusion:**

Mortality related to IMD is higher among children with severe meningococcemia despite early interventions in PICU. Routine use of meningococcal vaccines during childhood would be a better strategy for controlling IMD in both developing and developed countries.

## 1. Introduction

Invasive meningococcal disease (IMD) due to *Neisseria meningitidis* is an important cause of morbidity and mortality in children worldwide [1]. *N. meningitides* are classified into 12 serogroups according to the antigenic variability of their capsules, and among them, serogroups A, B, C, Y, W, and X are clinically important [1,2]. The epidemiology of serogroups changes over the years, and patient data on clinical presentation and laboratory workup will be helpful for diagnosis and treatment [1, 2]. A majority of the patients are below five years of age, and secondary peaks have been reported among adolescents and young adults [3]. While there are some risk factors associated with IMD, for example, travel to endemic regions, complement deficiency, splenectomy, HIV infection, or eculizumab use, the majority of the patients (>95%) with IMD were previously healthy [1, 2, 4].

Meningococcemia is a sudden onset, fulminant contagious disease requiring stay in an intensive care unit [5]. Despite the developments in supportive therapies and intensive care treatments, there is a high rate of mortality with IMD [1,2]. Within the last 20 years, case fatality rates (CFR) are reported between 9 and 12%, which varies according to serogroups and age, and CFR might increase in cases with meningococcemia, infections due to hyperinvasive clones, and in older age groups [6]. Herein, we report the clinical and laboratory findings of children with IMD requiring stay in a pediatric intensive care unit.

## 2. Patients and Methods

Patients who were admitted to the pediatric intensive care unit (PICU) of Sanliurfa Training and Research Hospital between January 2018 and June 2019 with a diagnosis of IMD were included. Diagnosis of IMD was confirmed by the detection of *N. meningitides* with culture and/or the polymerase chain reaction (PCR) method in cerebrospinal fluid (CSF) and/or blood samples. Patient's age, gender, presence of underlying disease, and vaccination status were recorded. Complete blood count, peripheral blood smears, serum C reactive protein (CRP) and procalcitonin levels, arterial blood gas analysis, phrothrombin time (PT), activated partial thromboplastin time (aPTT), fibrinogen, D-dimer, and blood culture results were noted from medical records. CSF workup results were recorded for patients who were eligible for a lumbar puncture. Patients were diagnosed as disseminated intravascular coagulation (DIC) if they met the criteria for at least one of the following: platelet count <150000/mm^3^, fibrinogen level <250 mg/dL, D-dimer level >500 U/L, long PT, and PTT (>13.2 seconds and >31 seconds, respectively) [7]. Septic shock was diagnosed with the criteria defined in “Surviving Sepsis Campaign: International Guidelines for the Management of Severe Sepsis and Septic shock: 2016” [7]. Pediatric risk of mortality (PRISM) scores were recorded for all patients with the worst clinical and laboratory data within the first 24 hours. Supportive extracorporeal therapies as renal replacement therapy and plasma exchange therapy were recorded. Vasoactive-inotropic score (VIS) was calculated with the formula: dopamine dose (*μ*g/kg/min) + dobutamine dose (*μ*g/kg/min) + 100 × adrenaline dose (*μ*g/ kg/ min) + 100 × noradrenaline dose (*μ*g/kg/min) + 10 × milrinone dose (*μ*g/kg/min) + 10,000 × vasopressin dose (U/kg/min). Prognosis of the patients has been classified as “died” or “discharged.”

## 3. Results

Twelve children (five girls and seven boys) aged between 5 and 168 months with IMD were enrolled. All patients were reported to healthcare authorities, and all of the persons with a history of close contact were started on prophylactic treatment by the Local Public Health Department. The clinical, demographical, and laboratory findings of the children are summarized in Tables 1 and 2.

Three patients were diagnosed with meningococcemia (cases 5, 6, and 12), while the remaining nine were diagnosed with meningococcemia and meningitis. Four of the patients in our study were younger than one year of age (33.3%), three were aged between 1 to 5 years, and five (41.6%) were older than five years. One child was a member of a Syrian refugee family. None of the patients had been previously vaccinated with meningococcal vaccines. Two of the patients (cases 4 and 10) had no history of other childhood routine immunizations.

PRISM scores vary between 5 and 37, and predicted mortality according to PRISM scores was between 1.7 and 94.2% (Table 2). The highest predicted mortality according to PRISM was for cases 1, 2, and 4 (88.2, 85.2, and 94.2%, respectively); cases 1 and 4 died (Table 2).

Fever was the common presenting symptom for all the patients. The period time between the onset of symptoms and presentation varied between 12 and 72 hours, and all of the patients were referred because of fever and petechiae/purpuric lesions. Cases 3, 6, 11, and 12 presented with petechiae and purpuric lesions on extremities, case 7 had purpuric lesions only in the anal region, and the rest of the cases had lesions disseminated over the entire body.

Five out of 12 patients had IMD due to serogroup B, and one case was due to serogroup W. All of the patients were given fluid resuscitation and antibiotic treatment of ceftriaxone (100 mg/kg/day 2 times or cefotaxime 200 mg/kg/day 4 times). The first dose of antibiotic treatment was given at the first hour of admission to the pediatric emergency care unit. Vancomycin was empirically given to case 4 due to suspected toxic shock syndrome until the causative bacteria had been identified (Table 2). Seven patients had signs of septic shock and DIC (cases 1, 2, 3, 4, 7, 9, and 12). Four cases were intubated and treated by mechanical ventilation support (cases 1, 4, 7, and 9). Cases 1 and 4 had continuous hemodiafiltration therapy, and case 9 had therapeutic plasma exchange (PLEX). Supportive therapies are summarized in Table 2 for these 12 patients who were treated with the diagnosis of IMD. All of the cases had an echocardiographic evaluation, and ejection fraction was found to be lower than normal in cases 1, 2, 4, and 9. Cases 1 and 4 went into catecholamine-resistant shock and did not survive despite treatment with stress doses of hydrocortisone, adrenaline, dopamine, and milrinone infusions. Case 1 was intubated with respiratory distress after seizures on day one of PICU admission and had diabetes insipidus within the next 48 hours. The patient died on day 8 after admission. Case 4, who was born as a twin, had growth retardation (body weight was <3^rd^ percentile) and a history of being treated for a symptom of fever within the previous two days. The patient presented with a history of extension posturing and convulsions at home, and purpuric lesions appeared within two hours of admittance. Purpura fulminans developed within hours, and the patient died on the fifth day of PICU admission.

## 4. Discussion

IMD continues to be an important public health issue that results in high rates of mortality and morbidity in childhood. Beginning treatment promptly and extensive therapy protocols are life-saving in these critically ill patients [1, 2, 8, 9]. In this study, we followed up 12 children with IMD in PICU, and two of these patients did not survive despite being treated early. Early antibiotic treatment (first hours of admission) is a crucial step in the treatment of meningococcemia. The targeted therapy approach should include not only starting antibiotic treatment immediately but also early recognition and treatment of complications of the infection such as shock [7, 8]. In our case series, four cases with catecholamine-resistant septic shock (VIS scores of 80, 85, 55, and 70, respectively), required stress doses of hydrocortisone. Stress dosages of hydrocortisone are indicated if the shock is exacerbated by adrenal insufficiency [8]. Dialysis or hemofiltration may be needed to compensate for kidney injury and to reduce the massive edema from fluid resuscitation [8]. Continuous veno-venous hemodiafiltration was performed in case 1 due to acute kidney injury, anuria, and fluid overload exceeding 15% for two days, and in case 4 due to cytokine removal for one day. We preferred this renal replacement modality because the blood pressure of our patients was not stable. We used PLEX with fresh frozen plasma four times in case 9 with sepsis-associated organ dysfunction with thrombocytopenia-associated multiple organ failure. We observed diabetes insipidus as an early period complication in one case with meningococcal meningitis. Meningococcal meningitis may be complicated by raised intracranial pressure or the syndrome of inappropriate antidiuretic hormone secretion [10]. Despite all treatment modalities, the mortality rate was 16.6% in PICU. In our study, two patients, who had laboratory and clinical findings of multiorgan failure, died. The rate of mortality was higher than the classic 10% mortality rate [6, 8]. We enrolled only children with severe conditions requiring PICU, and the predicted mortality regarding pediatric risk of mortality at admission was higher than 20% in seven out of 12 cases.

Recognition of symptoms is of critical importance in diagnosing IMD. IMD should be in the differential diagnosis in every pediatric case presenting with fever and petechiae [11]. Definitive diagnosis is confirmed by cultures of blood and CSF. Also, bacteria can be isolated from biopsies of skin lesions. In recent years, detecting bacterial DNA by PCR has become an important method of diagnosis in meningococcal diseases [12]. Bacteria were identified, and serogroups were defined in serum specimens in six patients. Serogroup B was the predominant serogroup in our case series. Even though blood cultures are positive in 40% to 75% of cases according to the literature, no growth was detected in any of our patients' blood cultures [13]. Two patients died in our case series, one due to serogroup B and the other due to serogroup W. New hyperinvasive clones of serogroup W have been admitted with severe clinical courses, and the case fatality rate of serogroup W is higher than that of serogroups B and C [8].

*N. meningitidis* has become the most important target agent of vaccine studies after successful implementation of vaccines, resulting in decreased prevalence of the other two most common agents causing meningitis, *Haemophilus influenzae tip B*, and *Streptococcus pneumoniae* [14]. None of the patients in our study had been vaccinated with meningococcal vaccines. Immunization against IMD is the best prevention strategy for controlling the disease. In Turkey, quadrivalent conjugated meningococcal vaccines (MenACWY) and meningococcal B vaccine (4CMenB) are available in private practice and are not part of the National Immunization Program. MenACWY vaccines have been routinely used in Hajj and Umrah pilgrimages and also have been used among military personnel [1]. Meningococcal conjugate vaccines and MenB vaccines have been routinely recommended for patients undergoing splenectomy and also patients requiring eculizumab treatment. Seroepidemiology of IMD in Turkey is dynamic and quite different when compared to other countries [14]. Serogroup B is the predominant strain, followed by serogroup W; other serogroups (A, Y, C, and X) have been sporadically reported [14]. IMD and serogroup surveillance are crucial for further vaccine implementation into the National Immunization Program.

Our study has some limitations. This is a retrospective, short-term interval, single-center experience and includes only children requiring PICU. We did not show the serogroup in six out of 12 cases. However, we report 12 cases with IMD from the city with the largest birth cohort, and our study results highlight the severity of the infection among previously healthy children. We underline the importance of early antibiotic treatment accompanied by early and intense supportive therapy in PICU, and these interventions could decrease the predicted mortality rate at admission. Routine use of meningococcal vaccines during childhood would be a better strategy for controlling IMD in both developing and developed countries.

## Figures and Tables

**Table 1:** Demographical findings, glasgow coma scale, and laboratory findings of 12 children with IMD.

Case number	Age (months)	Gender	GCS	WBC (mm^3^)	Platelet (mm^3^)	Lactate (mmol/L)	CRP (mg/dl)	PCT (ng/mL)	Echocardiography	Severity of sepsis	VIS	Nm Serogroup
1	72	G	3	33,290	20,000	8.3	170	100	EF 40%	MOSF	80	W
2	11	G	7	27,000	29,000	5.3	469	100	Normal	Septic shock	30	B
3	84	B	7	30,000	80,000	3	130	100	Normal	Septic shock	—	—
4	12	B	3	25,000	21,000	24	332	100	EF 30%	MOSF	85	B
5	62	G	10	10,300	73,800	2.8	69	40	Normal	Sepsis	—	—
6	60	B	7	18,000	47,000	3.6	86	45	Normal	Sepsis	—	—
7	26	B	7	33,000	30,000	3.8	157	100	EF 50%	Septic shock	55	B
8	56	B	12	21,500	65,000	2	76	80	Normal	Sepsis	—	B
9	168	B	9	35,690	12,000	5.9	180	100	EF 42%	TAMOF	70	—
10	6	G	13	19,100	66,700	1.5	82	55	Normal	Sepsis	—	B
11	70	G	11	23,000	98,000	1.7	74	85	Normal	Sepsis	—	—
12	11	B	11	13,500	74,000	2.0	70	50	Normal	Septic shock	—	—

PRISM: pediatric risk of mortality; GCS: Glasgow Coma Scale; PICU: pediatric intensive care unit; WBC: white blood cell; CRP: C-reactive protein, PCT: procalcitonin; VIS: vasoactive-inotropic score; G: girl B: boy.

**Table 2:** Clinical interventions in PICU, PRISM score, predicted mortality, and prognosis of 12 children with IMD.

Case number	Antibiotic	Fluid resuscitation at admission 20 mg/kg in 20 minutes (times)	FFP	Platelet	ECT	Mechanical ventilation	PRISM score	Predicted (%)	Length of PICU stay (days)	Prognosis
			Transfusion							
1	Cefotaxime	2	5	4	CVVHDF	MV	34	88.2	8	Died
2	Cefotaxime	2	2	2	—	—	32	85.2	7	Discharged
3	Cefotaxime	2		2	—		20	25.7	5	Discharged
4	Cefotaxime, vancomycin	2	4	4	CVVHDF	MV	37	94.2	5	Died
5	Cefotaxime	2	3	—	—	—	15	11.7	4	Discharged
6	Cefotaxime	2	3	—	—	—	13	8.2	3	Discharged
7	Cefotaxime	2	2	2	—	MV	25	56.7	3	Discharged
8	Ceftriaxone	1		—	—	—	5	1.7	3	Discharged
9	Cefotaxime	3	2	3	PLEX	MV	30	64.2	9	Discharged
10	Ceftriaxone	1		—	—	—	11	7.3	4	Discharged
11	Ceftriaxone	1		—	—	—	12	6.4	4	Discharged
12	Cefotaxime	2	3	2	—	—	17	21.1	4	Discharged

FFP: fresh frozen plasma, ECT: extracorporeal treatment; CVVHDF: continuous veno-venous hemodiafiltration, PLEX: therapeutic plasma exchange; PRISM: pediatric risk of mortality; PICU: pediatric intensive care unit.

## Data Availability

Access to data is restricted. Data sharing is not possible, as they include retrospective patient records, to protect the privacy of patient data.
